# Proof of Concept of Home IoT Connected Vehicles

**DOI:** 10.3390/s17061289

**Published:** 2017-06-05

**Authors:** Younsun Kim, Hyunggoy Oh, Sungho Kang

**Affiliations:** Department of Electrical and Electronic Engineering, Yonsei University, 03722 Seoul, Korea; faithful@soc.yonsei.ac.kr (Y.K.); kyob508@soc.yonsei.ac.kr (H.O.)

**Keywords:** connected vehicle, virtual personal assistant

## Abstract

The way in which we interact with our cars is changing, driven by the increased use of mobile devices, cloud-based services, and advanced automotive technology. In particular, the requirements and market demand for the Internet of Things (IoT) device-connected vehicles will continuously increase. In addition, the advances in cloud computing and IoT have provided a promising opportunity for developing vehicular software and services in the automotive domain. In this paper, we introduce the concept of a home IoT connected vehicle with a voice-based virtual personal assistant comprised of a vehicle agent and a home agent. The proposed concept is evaluated by implementing a smartphone linked with home IoT devices that are connected to an infotainment system for the vehicle, a smartphone-based natural language interface input device, and cloud-based home IoT devices for the home. The home-to-vehicle connected service scenarios that aim to reduce the inconvenience due to simple and repetitive tasks by improving the urban mobility efficiency in IoT environments are substantiated by analyzing real vehicle testing and lifestyle research. Remarkable benefits are derived by making repetitive routine tasks one task that is executed by a command and by executing essential tasks automatically, without any request. However, it should be used with authorized permission, applied without any error at the right time, and applied under limited conditions to sense the habitants’ intention correctly and to gain the required trust regarding the remote execution of tasks.

## 1. Introduction

As emerging technologies from the consumer electronics and IT technology fields crossover to the automotive domain, modern vehicles are being equipped with powerful sensors and networking and communication devices that can communicate with other vehicles and exchange information with the external environment [1,2,3]. A connected vehicle is evolving to have devices that can be connected to other devices within the vehicle itself and/or devices, networks, and services outside the vehicle, including other vehicles, the home, office, or surrounding infrastructure. The way in which we interact with our vehicles is rapidly changing, driven by the increased use of mobile devices, cloud-based services, and advanced automotive technology [4,5,6,7]. In particular, the machine interface between automobiles and humans must allow for the seamless integration of several types of personal devices that support various software and hardware standards to allow drivers to use their smartphones while driving. Therefore, the future vehicle will have the capability of surround sensing, and can form connections between vehicles, as well as between vehicles and surrounding infrastructure. This will lead to increased requirements for information and communication technology, and ultimately, cars will become a part of the Internet in the near future [8].

The future mobility of the automotive industry requires new applications and technologies related to electric powering, automation, and connected services [9]. Internet-integrated vehicles are already on the roads, and it is predicted that the percentage of internet-integrated vehicle services will jump from the current figure of 10% to 90% by 2020 [10]. The advances in cloud computing and the Internet of Things (IoT) have provided a promising opportunity in vehicular software and services in the automotive domain. Academia and the automotive industry are responding by exploring reliable and efficient connectivity solutions [11,12,13,14]. In recent years, the demand for high-speed mobile internet services has dramatically increased; hence, the requirements and market demand for IoT device-connected cars will continuously increase. However, IoT-based vehicular data clouds must be efficient, scalable, secure, and reliable before they can be deployed on a large scale. Existing algorithms and mechanisms are unsatisfactory to meet the necessary requirements simultaneously.

IoT-based vehicular data clouds are expected to be the backbone of the system, with the goal of making driving safer and more enjoyable. However, research into integrating the IoT with vehicular data clouds is still in its infancy and the existing study into this topic is highly insufficient. To ensure that vehicular data clouds are useful, numerous services such as road navigation, traffic management, remote monitoring, urban surveillance, information, entertainment, and business intelligence need to be developed and deployed based on vehicular data clouds [15,16,17,18,19]. Numerous challenges still exist such as security, privacy, scalability, reliability, quality of service, and the lack of global standards [14,18]. Due to the complexity involved in implementing vehicular clouds and integrating various devices and systems with vehicular clouds, a systematic approach and collaboration among academia, the automobile companies, law enforcement, government authorities, standardization groups, and cloud service providers is needed to address these challenges. Though there are many challenges, IoT and cloud computing provide tremendous opportunities for technology innovation in the automobile industry and will serve as enabling infrastructures for developing vehicular data clouds [11].

One of the most important applications of IoT technology is the development of smart homes, smart offices, smart cars, etc., in a smart environment domain [20,21,22,23,24]. Smart home environments are becoming a part of our everyday life where different devices and technologies are deployed. In particular, the smart home application, which has recently gained more popularity, includes a set of connected sensors, appliances, and gadgets that can be used to control and monitor various household characteristics such as heating, lighting, and security [20,21]. There are two immediate driving forces bringing IoT connectivity to vehicles. One is for the connected vehicle to communicate, via the mobile network, with the connected house, and the other is to adjust any connected devices in the home remotely. The mobile network plays a significant role in reliably and securely transferring relevant data from the vehicle to the house. The IoT technology enables linking physical objects, such as different devices and sensors, with virtual objects, which exist through internet connections, with increased comfort and safety. A seamless communication from the home to vehicle and to other parts of life is now expected. This is leading to smartphone connectivity with the vehicle and the connectivity of the vehicle with the cloud, etc. However, limitations exist regarding the evolution of cellular connectivity once the necessary components are embedded in the vehicle [25].

Nowadays, there is a growing demand for continuous access to the Internet in vehicles to provide improved safety, comfort, mobility, and entertainment. As more people spend a considerable part of their lives in their vehicles, drivers will increasingly demand access to applications such as Google Maps and YouTube using the familiar interfaces in their car [4]. Additionally, as mobile devices continue to grow in usage, people are creating secondary interface experiences related to, but not necessarily attached to, their vehicles, making this type of device a prime choice for any vehicular application. Moreover, they expect to have the same connectivity in their vehicles as they have at home and at work. Therefore, future automotive IT infrastructure and interaction specifications should follow on from general human–computer interaction guidelines with the understanding that there will be a high heterogeneity required to manage these contexts [26].

Even when end users can communicate with the IoT via multiple modalities, one of the most common ways to interact with electronic devices is through a graphical user interface (GUI). However, graphical interfaces can be confusing and difficult to use when users employ different interfaces in several environments. As the main modality that humans use to communicate with each other, speech can be one of the most convenient means of interaction. In terms of vehicle safety [27], the user interface is designed with connectivity in mind, significantly enhancing the possibilities of such technologies as speech synthesis and speech recognition [28,29]. A recent advance in speech recognition is the adaptation of natural language understanding technology, thereby improving the efficiency of human-computer interactions [30]. Speech interfaces are attracting more attention, and therefore, speech recognition technology is becoming an integral part of interacting with IoT devices. In the near future, speech interface technology will enable the end users to naturally speak to their household devices instead of pushing the buttons or clicking the icons on a GUI. 

Vehicles have become a sophisticated and pivotal piece of the IoT and have always been an extension of us, integrated into our lifestyles and daily tasks as a reflection of our human experience. Thanks to Internet technologies, the vehicle is able to interact with other connected ecosystems such as the smart home, providing seamless mobility [31]. Drivers increasingly want to stay connected to their homes while driving and enable seamless home automation links to Internet-enabled smart devices, such as lights, home security systems, automatic garage doors, and more. They want to experience an interaction to open a garage door and to turn on a light on the dashboard of their vehicles or through voice control when they are a certain distance from home. Our research is conducted to illustrate the need of vehicle-to-home connectivity and to demonstrate it by providing drivers with a convenient and responsible way to remotely access all of their most frequently used smart home devices while on the road.

In this paper, we propose the concept of a home IoT connected vehicle, which is a vehicle connected to the home as a part of the IoT ecosystem. It has a voice-based virtual personal assistant comprised of a vehicle agent and a home agent, and always stays with a driver as a personal IoT partner to perform several kinds of activities while driving, at home, and at the office. It is implemented by merging an in-vehicle infotainment system and a cloud-based smart home platform. For the vehicle, the in-vehicle infotainment system, in which a smartphone is linked and the cloud-based smart home server and natural language processing server are connected, is developed and integrated into the vehicle. For the home, a smartphone-based natural language interface input device includes an Android-based smartphone and near-field noise-canceling microphone. It connects the same cloud-based smart home server and natural language processing server, providing the same services for the vehicle. The proposed concept is substantiated by analyzing real vehicle testing and lifestyle research through the home-to-vehicle connected service scenarios; the way to home, arriving at home, living at home, and leaving home. The paper is organized as follows: Section 2 presents the proof of concept for the home IoT-connected vehicle, and Section 3 describes how to design the home IoT-connected vehicle. Section 4 provides four categorized groups of home-to-vehicle connected services, and Section 5 provides the evaluation results. Finally, Section 6 concludes the proposed proof of concept.

## 2. Proposed Concept 

**Figure 1:**
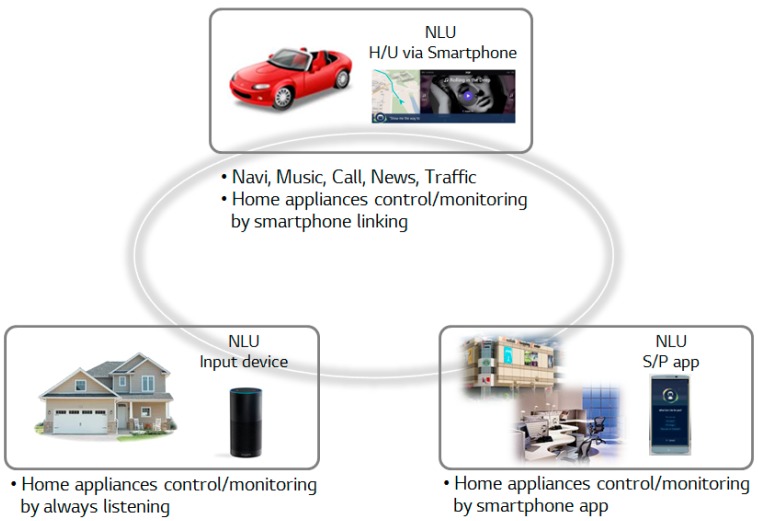
Concept.

The proposed concept is a home IoT connected vehicle with a voice-based virtual personal assistant and it is made up of a vehicle agent and a home agent, as shown in Figure 1. The virtual personal assistant always stays with a driver as a personal IoT partner and performs several kinds of activities to be able to do things like turn on the A/C, lock/unlock the doors, and turn on lights, as well as to support functions like playing music, making a phone call, and navigating while driving, at home and at the office. A user can communicate with it through the homogenous voice-based natural language interface, both in the vehicle and while at home. One interesting feature is to be able to manage their smart home directly and access all kinds of content provided by the smartphone, anywhere and at anytime. Another is to provide cloud-based personalized services within the home-to-vehicle connected environment using a unified speech interface based on natural language that is supported in different environments such as the home, vehicle, and office. This makes it possible for users to have the same connectivity in their vehicles as they have at home and at work.

The vehicle agent is not only an interpreter, but also a communicator, and interacts between a smartphone-linked in-vehicle infotainment system and smart home server. It can control and monitor home appliances from anywhere the driver wants to be and offers seamless communication from the home to vehicle and to other parts of life. It has a smartphone-based connectivity and manipulates IoT devices even while driving, if necessary. A cloud-based smart home server plays a major role for home IoT devices to be remotely monitored and controlled in the vehicular environment since it collects and distributes the information from and to the home and vehicles. It is also designed to be able to maximize the use of a smartphone in the vehicle. For this purpose, a smartphone provides the contents for music, calls, cloud services, and Google navigation. In addition, it offers a speech interface for connecting the vehicle to the driver through voice activation, voice recognition, and natural language understanding.

The home agent is a smartphone-based natural language input device and can control and monitor home appliances from anywhere the user chooses through the cloud-based smart home platform, which is composed of a smart home server and different types of home IoT devices. The natural language input device is made up of Android-based smartphones and near-field noise-canceling microphones. A high-performance microphone is also used to maintain accuracy and precision between the smartphone, signal strength, and other factors, since the voice signal conversion to text form depends on distance. In a smart home domain, personalization plays an important role in speech interactions with the devices used daily. In general, different households have different sets of connected devices. Therefore, a specialized voice activation technology and the inhabitants’ preferred keywords are applied to allow inhabitants to more naturally customize their speech communications with their devices. Specifically, personalized language models that recognize the customized device names are suggested, since it is important to accurately recognize the device name when each end user issues a personalized command for a device [32]. 

Apple’s Siri [33] and Microsoft’s Cortana [34] are smartphone-based intelligent personal assistants and they use a natural language user interface to answer questions, make recommendations, and perform actions. Additionally, Amazon’s Alexa [35] is an internet (Ex. Ethernet)-based intelligent personal assistant for the home and is also capable of voice interaction, music playback, making to-do lists, setting alarms, streaming podcasts, playing audiobooks, and providing weather, traffic, and other real time information, such as the news. However, current technology makes it difficult to directly control home IoT devices though the speech interface since the IoT ecosystem is not established yet. It is not easy to integrate all kinds of cloud-based servers, including the voice recognition engine, natural language processing engine, and cloud-based home server. In this research, we integrate the cloud-based Google voice recognition engine, the cloud-based LG natural language processing engine, and a specialized LG voice activation technology, and demonstrate home-to-vehicle connected service scenarios. In addition, allowing each end user to customize their speech communications with their devices creates a more natural interaction. Specially, it produces dramatic effects when personalized names for every object in their set of connected things are used. In our research, the post processing technology employed to be able to detect a predefined word to utilize the always listening status of the voice recognition engine and to support the selected names to issue voice commands is applied to be optimized for the inhabitants.

## 3. Home IoT Connected Vehicle

**Figure 2:**
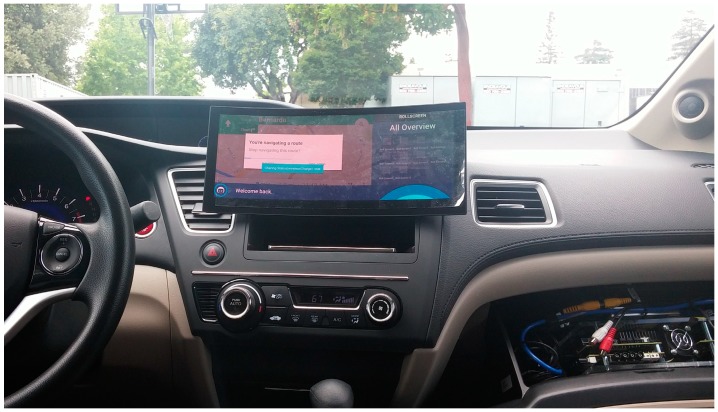
Home IoT connected vehicle.

The home IoT connected vehicle is implemented by merging an in-vehicle infotainment system and a cloud-based smart home platform. For the vehicle, the in-vehicle infotainment system, in which a smartphone is linked and the cloud-based smart home server and natural language processing server are connected, is developed and integrated into a HONDA Civic vehicle, as shown in Figure 2 [36]. For the home, a smartphone-based natural language interface input device including an Android-based smartphone and near-field noise-canceling microphone is developed. It connects the same cloud-based smart home server and natural language processing server and provides the same services for the vehicle. Smart appliances commercially available from manufacturers around the world are integrated into the lifestyle research house located in that state of California in the United States. The technologies that exist within the house are primarily used as a means of making a user’s life more comfortable and more interesting. This work is conducted in partnership with HONDA Silicon Valley Laboratory and the vehicle component technology center of LG Electronics.

### 3.1. In-Vehicle Infotainment System

**Figure 3:**
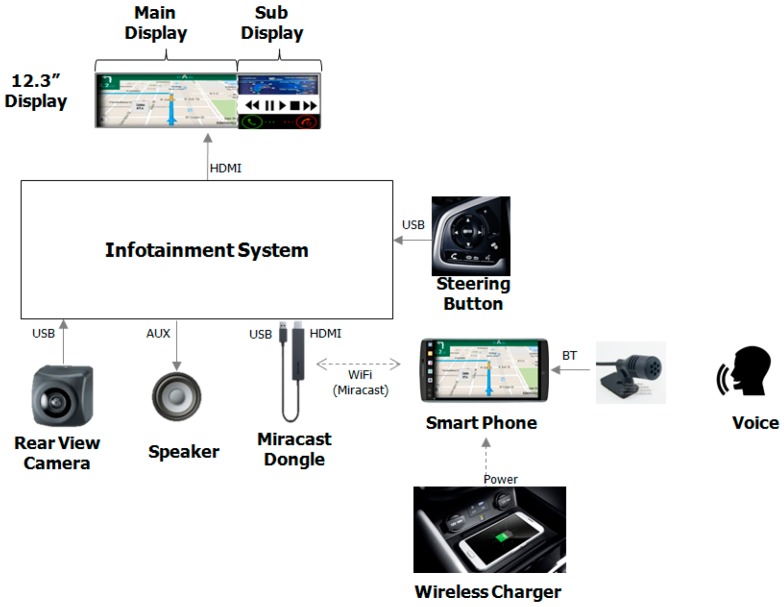
In-vehicle Infotainment System.

The in-vehicle infotainment system, which has the smartphone connectivity and vehicle interfaces such as a rearview camera sensor, in-vehicle speaker, and steering wheel button, is proposed as shown in Figure 3. An Android-based, smartphone-linked in-vehicle infotainment system with a 12.3-inch display and a bi-partitioned screen composed of a main shot and a sub shot is used. The display is configured to show the contents easily and naturally. It is designed such that each screen size can be controlled by multi-layered screen composition technology and the display contents are adjusted according to each substantiation scenario. The system can remotely monitor or control home devices and display the appropriate information coming from the smartphone on the dashboard display. The LG V10 smartphone is used for connecting the vehicle and driver for this test. The smartphone is capable of voice activation and recognition; it connects to a smart home platform for IoT devices and uses the LG natural language processing engine [37]. For the screen mirroring feature, the Miracast standard is used for a wireless connection from the smartphone to infotainment system through a dongle-type device [38]. For the vehicle interface, the buttons typically found on the steering wheel and a gearbox are used. Furthermore, a wireless charger is used to avoid rapid battery consumption. For the speech interface, a Bluetooth-based microphone is used to recognize the driver’s voice command and an in-vehicle speaker is used for the audio system of the vehicle. In general, the Android application converts the input voice command given on the smartphone to the text form. Then, this signal in text form is transmitted from the smartphone to a cloud-based smart home server. We have used the same technology for this paper. 

A real-time embedded system is applied using the nVIDIA Jetson TK1 reference board, the Ubuntu-based Linux is used as an operating system, and the GUI is implemented with the Qt programming tool, as shown in Figure 3 [39]. All of the applications comprising the smart home IoT control, including the real view camera image drawer, call, music, and mirroring-based Google navigation, are implemented using C & C++ programming languages. Gearbox information is obtained from the Arduino-based microcontroller platform that acts as an interface between the real vehicle and in-vehicle infotainment system [25]. Google navigation by smartphone mirroring is supported in a driving mode and image viewing for a real view camera is provided in a parking mode for the main display contents. The smartphone provides the contents of music, calls, cloud services, and Google navigation. Music, calls, news, and smart home IoT control functions are supported for the sub display contents. For the speech interface, the Google voice recognition engine and cloud-based NLU engine are used. Depending upon the voice commands given, the infotainment system performs the actions accordingly.

### 3.2. Cloud-Based Smart Home Platform

**Figure 4:**
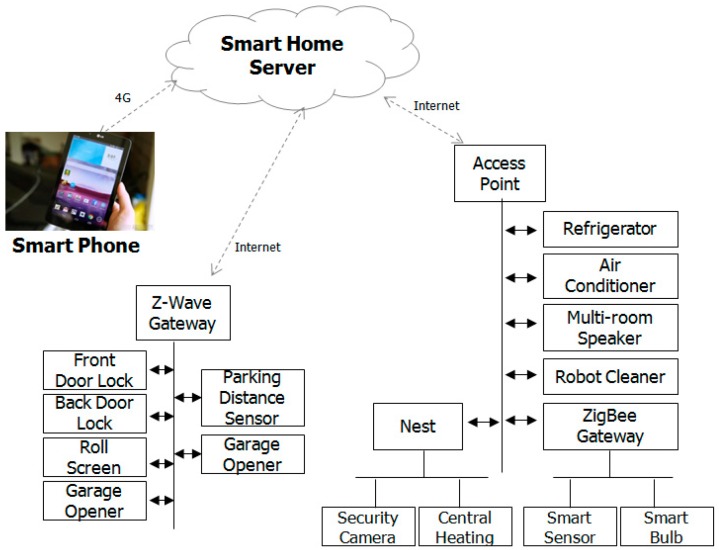
Home Devices connected to Smart Home Server.

The cloud-based smart home platform, which connects the vehicle to the home or vice versa by smartphone-based connectivity, is suggested as shown in Figure 4. All home IoT devices that could be integrated together in a unified platform for the maximum benefit and control include a robotic cleaner, an air conditioner, refrigerator, multi-room speaker, lighting, temperature control, and automatic parking control system. A cloud-based smart home platform is used for all digital appliances to be remotely monitored and controlled for both the vehicle and home environments, utilizing a homogeneous speech interface. Along with an increasing number of digital appliances in the smart home space, complex control or management, including numerous data storage systems, is becoming a heavy burden on the smart home system [40]. 

Cloud computing represents an on-demand service model for delivering resources ranging from storage and data access via computation to software provisioning, so it becomes an ideal alternative. Thus, we consider that the Smart Home can merge into the cloud to provide more services and obtain more information provided by the cloud. Zigbee and Wi-Fi wireless home automation technology are being adopted to modify existing housing. The coexistence of wireless technologies in IoT (Wi-Fi, ZigBee, Bluetooth, and Bluetooth Low Energy) is considered for an indoor environment [41,42,43,44]. As the number of devices increases, the possibility of interference in a home environment also increases. Interference in a home environment is a result of devices using different technologies for communication. Therefore, different types of gateways are used to connect different technologies within the home to provide access from the home to external services and vice versa.

As the grouped-controlled IoT devices for this study, 12 roll shades and eight smart bulbs are used. The roll shade device uses the Z-Wave communication protocol and can be connected to the Internet by a Z-Wave gateway device with an Ethernet interface. The smart bulb device has the Zigbee communication protocol and can be connected to the Internet by the Zigbee gateway device with a Wi-Fi interface. We have applied smart bulb devices as the lighting sources for a living room, a dining room, and a kitchen. The smart ThinQ sensor is used to detect the status of the washer and dryer using another Zigbee communication device [37]. The device detects the vibration of the washer and dryer and can distinguish the end of the operation. In addition, a refrigerator, an air conditioner, a robot cleaner, and two multi-room speakers are used as Wi-Fi communication devices [37]. A motorized garage door opener and an ultrasonic sensor, which can detect the parking distance when a car enters the parking lot and drives to a reserved parking space, are used for automated parking control. For the parking distance detection sensor, two ultrasonic sensors are used. If a vehicle is correctly parked in the parking lot, the light shows a green color; otherwise, the light shows a red color. Moreover, the status of the sensor is transferred to a cloud platform and used to automatically open the back door located in the parking lot.

## 4. Home-to-Vehicle Connected Scenarios

The proposed concept is substantiated through the home-to-vehicle connected service scenarios, which are described in Table 1, Table 2, Table 3 and Table 4: the way to home, arriving at home, living at home, and leaving home. It is suggested, as a means for the driver, for users to make use of the IoT in their daily living. We use both the lifestyle research method and the real vehicle testing method to prove the concept and to ensure that our study is as realistic as possible. We assume that the driver is a commuter and periodically visits several locations since it is quite easy to find repetitive lifestyle patterns, both before starting to drive and after driving. The possible service scenarios of a driver making use of the IoT in their daily living are suggested as primary and are designed to reduce the inconvenience of simple and repetitive tasks by improving the urban mobility efficiency. It uses the vehicular data cloud in an IoT environment that has the capability to integrate numerous devices available within the home and devices in the vehicle. Therefore, we focus on how cloud services could be effectively utilized for the real lifestyle when an end user rides in a vehicle with devices connected through the IoT. 

The first scenario is the way to home. In this scenario, the smartphone is placed on the wireless charger and the welcome message is automatically shown whenever the driver gets into the vehicle. When a driver is near his or her vehicle, the door unlocks and is automatically opened. Moreover, the status information will be sent to the IoT cloud. It is important to note that these sensors can be freely placed all around the doors and doorjambs since they are relatively inexpensive. We believe that they will be triggered by an input from the pressure or force sensors or other types of sensors around the doors and doorjambs. A hypothetical scenario could be the enabling and integration of wireless sensor networks and the IoT environment. The cloud-based smart home platform can then determine the time to transmit every control command.

The second scenario is for arriving at home. As a driver arrives home, the vehicle receives the GPS information and the voice message is given to the driver to trigger the garage door opener remotely. When the driver gives the voice-based command, while in the vehicle, the garage door is opened remotely and automatically. When the vehicle is detected after entering into the garage, the garage door is closed automatically by the command from the smart home server. This action is triggered by the parking distance detection sensor. The use of parking distance sensing with two ultrasonic sensors is designed to detect when the vehicle enters the parking lot and heads to the correct reserved parking space. When the vehicle is parked in the given space, the smart home server automatically transmits a command to open the backdoor lock located inside the garage.

The third scenario is for living at home. This scenario assumes that repetitive lifestyle patterns are conducted automatically to minimize routine tasks. Sequential commands from the cloud-based smart home server are given to turn on the lights, play music from multi-room speakers, and open the grouped 12 roll shades. The scenario assumes that the roll shades are initially closed, and the lights are dimmed prior to detecting any illumination changes. All types of IoT devices are controlled when voice commands are received from the habitant. Room illumination controllers were deployed in an actual house using smart bulbs. In addition, seamless music playing was conducted by multi-room speakers. The refrigerator and air conditioner could also be controlled using the temperature information provided by the Nest built in the house. 

The last scenario is for leaving home. This scenario assumes that repetitive lifestyle patterns are conducted when the driver or habitant leaves the home for the same purpose of minimizing routine tasks. The light bulbs and multi-room speakers are turned off and roll shades are automatically closed by the opening of the front door lock. This operation is done after the status of all IoT devices is checked. When the front door lock is closed, the status of the IoT devices is automatically transferred to the smartphone, allowing the driver to verify the proper operation. 

## 5. Experimental Results

In our research, real vehicle testing is conducted for the way to home and arriving at home scenarios and virtual home life experience testing is accompanied for the living at home and leaving home scenarios. For vehicle testing, a 5 km driving route on an urban road, which starts from the office and ends at home, is used to validate the way to home scenario and arriving at home scenario. The route guidance is given from the Google navigation and the participants issue voice commands and check the status according to each command while driving. For safety, participants are instructed to drive under a speed of 60 km/h. A lot of efforts are needed to enhance the performance of voice activation and voice recognition. Specially, a huge acoustic database collection is necessary and an elaborate training procedure is required to support the specific keyword recognition by anyone. For home testing, it is confirmed that each IoT device works correctly and properly according to the voice command in Standalone. As the next step, interoperability testing is performed through various other wireless routers. The unreliability issue of voice recognition exists according to the distance between the user and natural language interface input device. It is discovered that the recognition accuracy needs to be improved. In addition, system operational tasks such as new device registration, device initialization, and failure monitoring, etc., need to be tightened up to stabilize the smart home server.

Table 5 summarizes the evaluation result for the home-to-vehicle connected service scenarios mentioned in Section 4. According to the study, since almost all of the tasks of the first scenario to do things like lock/unlock the door, turn on the A/C, and receive today’s schedule are very routine, not so essential, and preferable, it is hard to expect the greatest effectiveness when it is served automatically or controlled remotely. Above all, it is insufficient to give simple information such as the current temperature or today’s schedule for users to feel comfortable and useful. More customized offerings need to be provided to meet their expectation based on the user’s preferred recommendations.

The experimental result of the second scenario indicates that a special benefit can be derived since it makes repetitive routine tasks one task, executed by a command. When the driver approaches home, a voice command for opening the garage door is given and it is used as a triggering event for the other sequential tasks. It is generated by detecting the location of the house and the remaining tasks of the scenario are executed automatically in the perfect order. The driver is not forced to get out of the car and he can open and close the garage door and open the back door of their house without any action. It can substitute routine and repetitive works for a human being. Of course, it needs to be provided at an appropriate time and with no fault. However, there is the possibility that the current voice command can be replaced with another method and user convenience can be maximized when IoT devices are fully connected in the near future.

In relation to the third scenario, we realize it is useful to utilize the control command by analyzing the data base acquired from the cloud-based home server. Since it collects and distributes the information from and to the home and vehicles, it predicts personalized preferences and creates useful applications in various ways. In this research, a limited number of devices are integrated and the scenario is proposed to only apply essential tasks executed automatically without any request, even if not repetitive. However, some scenarios have the capability to integrate numerous devices, as much as possible. For this purpose, it is important that the smart home platform is designed to allow for expansion in the scope and service to offer special and efficient home services for different types of digital appliances. Control commands should be applied without any error, at the right time, and with authorized permission. Otherwise, users feel significant inconvenience and will no longer use these services. In addition, since the status of each device can be changed at any time by anyone, this can cause a serious security situation or issue. For example, a garage door can be opened or the music can be played from the multi-room speaker in the middle of the night. Regarding the natural language interface for home scenarios, we also find it is very important to maximize the accuracy and precision, since a voice signal’s conversion into text form depends on the recognition distance.

The fourth scenario test results show that it is important to sense the intention of habitants’ departure correctly as part of the process of gaining trust for their delegating work. It is assumed that there are no remaining users in the house and the user goes out for the purpose of being away for a long time. The status of grouped IoT devices is changed by the status transition of the front door lock. It is necessary to verify when control commands are executed and the changed status is transferred to habitants. The ability to receive the status of IoT devices remotely makes the user feel secure, even if they do not check the status of the home in advance before leaving. This is adapted in reality when the user does not have enough time to check the status or forgets to check it.

## 6. Conclusions and Future Work 

In this paper, we propose the concept of a home IoT connected vehicle with a voice-based virtual personal assistant. The virtual personal assistant stays with a driver to consistently assist while driving, at home, at the office, or while running errands. The system provides a communication channel between the user and the virtual personal assistant using a homogeneous voice-based natural language interface, both in the vehicle and at home. The proposed concept was evaluated by implementing a linked smartphone and home IoT devices that were connected to a vehicle’s infotainment system. In addition, a smartphone-based natural language interface input device and cloud-based home IoT devices for the home were utilized. Several home-to-vehicle connected service scenarios were substantiated with the aim of reducing the inconvenience of simple and repetitive tasks by improving the urban mobility efficiency in an IoT environment by performing real vehicle testing and lifestyle research. The scenarios included the following: the way to home, arriving at home, living at home, and leaving home. This study shows that it is insufficient to provide only simple information such as the current temperature or today’s schedule. A remarkable benefit is derived by combining sequentially repetitive routine tasks into one task or by executing essential tasks automatically, without any request. However, the automatic execution of tasks should be used with authorized permission, applied without any error only at certain fixed times, and applied under limited conditions to sense habitants’ status correctly. This will allow the habitants to gain trust regarding the remote execution of tasks.

Future work may include more comprehensive study in relation to driving safety and vehicle security. The connected car will be more entertaining, more efficient than traditional cars, and an advanced IT technology such as IoT will provide new functionalities, including the home-to-vehicle connected services proposed in this research. However, it will inevitably increase the driver’s in-vehicle interactions and decrease their attention whilst driving, potentially causing additional driver distraction while driving. In order to mitigate driver distraction while using mobile devices, a safer interaction design should be considered under the circumstance of driving since it is important to consider safety first over convenience [27,45]. The connected car is an interim step on the way to the truly autonomous vehicle, which will free up drivers to conduct other activities while en route to work and during trips. In this step, finding an efficient way to optimize drivers’ current in-vehicle use is meaningful and practical in terms of road safety and we believe that a speech interface will certainly become an increasingly vital part of how we interact with our cars [46]. 

Securing the connected car from cyber-attacks is no easy task. Additionally, the wealth of data available about the cars and its occupants—their whereabouts, driving habits, behavior, preferences, and interests—will need to be carefully managed, to keep it private and to decide who gets to use it and how [45]. Eventually, a cloud will benefit from IoT by increasing its scope to deal with existent world things, providing a significant number of new services for real-life applications such as smart city [47], smart surveillance [48], etc. However, a very reliable security infrastructure, including methods for users to authenticate, use a two-way mapping process between users and devices, access policy creation and enforcement, logging and alerting capability, and secure communication should be considered above all things. The security challenges for the integration of IoT and cloud computing, and the secure integration of mobile devices into the vehicle must be considered for the direction of future research [49,50,51].

## Figures and Tables

**Table 1:** Scenario for an individual on the way home.

Event	Triggering and Response
Welcome Message	Door unlock and open
Traffic Information	Engine start and receive updated traffic information
Temperature control	Receive the current temperature and control the air conditioner
Remote control of air conditioner	Control the air conditioner in the house
Schedule Information	Receive today’s schedule

**Table 2:** Scenario for arriving at home.

Event	Triggering and Response
Welcome Message	Receive GPS information
Remote control of garage door	Open the garage door
Parking information	Detect vehicle entering garage
Remote control of garage door	Close the garage door
Remote control of door lock	Open the back door lock

**Table 3:** Scenario for living at home.

Event	Triggering and Response
Welcome Message	Receive the back door lock status
Remote control of grouped IoT devices	Close the back door lock, turn on the lights, music player and open the roll shade
Illumination information	Detect the illumination change or receive the control command
Remote control of grouped IoT devices	Close the roll shade and dim the lights

**Table 4:** Scenario for leaving home.

Event	Triggering and Response
Sense the departure	Receive the leaving home message
Remote control of grouped IoT devices	Open the front door lock, turn off the lights and music player, close the roll shade
Remote control of door lock	Close the front door lock
Home information	Receive the status of IoT devices

**Table 5:** Evaluation Summary.

Scenarios	Task Characteristics	Implications
Way to home	Routine, not so essenstial, preferable	It is insufficient to give a simple information such as current temperature or today’s schedule for users feel comfortable and useful.
Arriving at home	Routine, sequential, repetitive	It can substitute routine and repetitive works for a human being.
Living at home	Not routine, essential, repetitive	It is useful to utilized the control command by analyzing data base acquired from cloud-based home server.
Leaving home	Routine, sequential, repetitive, conditional	It is important to sense the intention of habitants’ departure correctly as part of the process of gaining trust for their delegating work.
